# Bispecific T cell engagers control solid tumors through clonal replacement and IL2-driven effector differentiation of CD8 T cells

**DOI:** 10.64898/2025.12.04.692214

**Published:** 2025-12-06

**Authors:** Matthias Obenaus, Clara L. Poupault, Christopher S. McGinnis, Céline Prange, Hua Jiang, Leon L. Su, Xiaojing Chen, Zhuang Miao, Joseph J. Muldoon, Winnie Yao, Deepa Waghray, Qinli Sun, Justin Eyquem, Rogelio A. Hernández-López, Ansuman T. Satpathy, Julien Sage, K. Christopher Garcia

## Abstract

Bispecific T cell engagers (TCEs) often exhibit limited efficacy in solid tumors, in part due to immunosuppressive cues in the tumor microenvironment and low expression of targetable tumor antigens. Therapeutic strategies to improve TCE target sensitivity and enhance T cell effector functions therefore have significant translational potential. Here, we engineered TCEs that induce T cell activation *in vitro* against the low-abundance target antigens, TRP2/K ^b^ and DLL3. Despite *in vitro* activity in these models, TCE monotherapy showed limited control of tumor growth in immunocompetent mice. Leveraging this *in vivo* model of TCE treatment failure, we discovered that co-treatment with TCE and a CD25-biased Interleukin-2 (IL2) rescues anti-tumor activity. Further, multimodal single-cell transcriptomic and immune repertoire analyses revealed that TCE-IL2 combination therapy controlled tumors by recruiting and activating new CD8 ^+^ T cells into the tumor microenvironment. These findings demonstrate that TCE-mediated anti-tumor responses function through a CD8 ^+^ T cell clonal replacement mechanism that can be augmented by cytokine therapy.

## Full Text Availability

The license terms selected by the author(s) for this preprint version do not permit archiving in PMC. The full text is available from the preprint server.

